# Chronic effects of different exercise types on brain activity in healthy older adults and those with Parkinson’s disease: A systematic review

**DOI:** 10.3389/fphys.2022.1031803

**Published:** 2022-11-28

**Authors:** Leilei Wang, Feiyue Li, Lu Tang

**Affiliations:** ^1^ Faculty of Physical Education, Pingdingshan University, Pingdingshan, China; ^2^ School of Exercise Science and Health, Dalian University of Technology, Dalian, China; ^3^ Department of Physical Education, Civil Aviation Flight University of China, Guanghan, China

**Keywords:** Parkinson’s disease, healthy older adults, brain activity, Tai Chi, treadmill, dancing, exercise

## Abstract

**Objective:** This study aimed to compare the regulation of brain activity by different kinds of long-term exercises (Tai Chi, treadmill training, and dancing) in healthy older adults and those with PD.

**Methods:** From January 2000 to October 2021, the electronic databases PubMed, Web of Science, and Scopus were searched. All articles were screened throughout the inclusion and exclusion criteria, which was followed by PICOS criteria. Finally, all articles were systematically reviewed with analyses.

**Results:** 29 studies were identified for this review, 24 of which were finally included in a group of healthy older adults, and five of which in a group of people with PD. All studies showed that significant changes were showed on people with PD and healthy older adults’ brain activity after three terms of the exercises we chose. An inverse change trend on the functional connectivity in people with PD was observed after treadmill training, whereas increased brain activity, cognitive function, memory, and emotion were noticed in healthy older adults.

**Conclusion:** Our findings suggest that different patterns of brain activity were also observed between healthy older adults and people with PD after treadmill training. However, more robust evidence and comprehensive studies are needed to determine if there is a difference between healthy older adults and people with PD.

## 1 Introduction

Parkinson’s disease (PD) has become the second-most common neurodegenerative disorder that typically affects older adults (Hirtz et al., 2007; Wirdefeldt et al., 2011). The main characteristic clinical hallmarks are the degeneration and loss of primarily dopaminergic neurons in the *substantia nigra*, and the accumulation of misfolded intracellular alpha-synuclein (α-syn) within Lewy bodies (Balestrino and Schapira, 2020). PD leads to the reduction of dopamine secretion and degeneration of the nigrostriatal pathway. With disease progression, the “Lewy body disease” will spread to the neocortex and cortical areas (Tysnes and Storstein, 2017). Recent research shows that the risk of developing PD is associated with genetic factors, consumption of dairy products, history of melanoma, and traumatic brain injury (Ascherio and Schwarzschild, 2016). Meanwhile, pollution due to pesticides and heavy metals resulting from industrialization is also a potential factor (Li et al., 2021). The classical clinical signs of PD are divided as follows: motor symptoms (i.e., bradykinesia, tremor, and rigidity); and non-motor symptom (i.e., cognitive impairment, sleep disorders, and depression). These co-occurring and prevalent symptoms of PD will induce serious psychological or medical pathology and incur a huge burden on society due to deterioration of physical health and loss of productivity (Silva De Lima et al., 2019). Globally, approximately 1% of all older adults aged 65 years old or above suffers from PD, and this percentage is predicted to increase with the growth of the aging population (Moore et al., 2005; Kasten et al., 2007; Lees et al., 2009). Current regimens in the treatment of PD are conventional and permanent, including drug treatment (especially levodopa) and surgical treatment (Ashoori et al., 2015; Mico-Amigo et al., 2017). Mainly, the motor symptoms of the disease are alleviated by these treatment approaches. However, the long-term use of drugs can lead to severe side effects, such as drug dependence and loss of efficacy.

Physical exercise can be used as an important supplementary treatment to improve these symptoms in people with PD. For example, treadmill walking exercise (Herman et al., 2007; Fisher et al., 2008) or progressive resistance training (Brienesse and Emerson, 2013; Lima et al., 2013) can improve these motor symptoms (i.e., muscle strength and endurance, mobility, and spatial parameters); and mindfulness yoga (Kwok et al., 2019) or resistance training reduces depressive, anxiety symptoms and improves the quality of life and functionality of older adults with PD (de Lima et al., 2019). Furthermore, many studies have also reported the benefits of exercise for others; it can enhance the plasticity of the nervous system for older adults (Cotman and Berchtold, 2002; Erickson et al., 2007) and people with brain injury (Betker et al., 2007), mild cognitive impairment (Hemmeter and Ngamsri, 2022), and Alzheimer’s disease (Zhou et al., 2022).

Different exercise modalities, such as endurance training, Tai Chi, whole-body vibration (Dincher et al., 2019), water exercises (Loureiro et al., 2022; Peyre-Tartaruga et al., 2022), and dancing, are feasible for healthy older adults and those with PD. For instance, long-term endurance training may even increase the cortical volume of the prefrontal area and the connectivity between brain regions, resulting in better emotion, memory, attention, and executive control abilities (Weng et al., 2017). Similarly, the research finding supports the Tai Chi group which demonstrated stronger frontostriatal functional connectivity in trials (Liu et al., 2020). Moreover, Rektorova et al. (2020) found that changes in the brain structure were observed after dance training compared with the control group; moreover, the executive functions slightly improved.

In parallel, previous works showed that changes in the brain structure and connectivity with ageing and PD impact cognitive processes, walking, and balance (Seidler et al., 2010; Tian et al., 2017), likely due to the involvement of common neural centers (Stuart et al., 2018). Specifically, some changes in certain brain areas (such as gray matter atrophy, changes in brain function connections, etc.) may cause movement disorders (Yogev-Seligmann et al., 2008; Agosta et al., 2014; Sehm et al., 2014; Hoffstaedter et al., 2015). Therefore, these three forms of exercises (Tai Chi, treadmill training, and dancing) may improve motor and non-motor symptoms by changing brain activity and structure.

Recent studies on the brain activity of exercises have not generally focused on neurological mechanisms, but those which did have mainly utilized electroencephalography (EEG), functional magnetic resonance imaging (fMRI), and functional near-infrared spectroscopy (fNIRS) (Chang et al., 2017). Innovations in non-invasive neuroimaging have been able to make remarkable advances in rapidly assessing cortical activity for healthy older adults and patients with PD.

Although evidence supporting the use of Tai Chi, treadmill training, and dancing to improve clinical measures of motor and cognitive functions exists, most studies were limited to focusing on the effects of exercise intervention and lacked the comparison between some exercise interventions. To the best of our knowledge, several systematic reviews have systematically investigated the effect of single exercise interventions, such as dancing, endurance training, and Tai Chi (Halloway et al., 2017; Pan et al., 2018; Pereira et al., 2019; Muinos and Ballesteros, 2021). However, it is unclear if the effects between these three types of exercises, healthy older adults, and people with PD are different. Therefore, this review aimed to compare the effects of the different kinds of exercises (Tai Chi, treadmill training, and dancing) on brain activity in healthy older adults and patients with PD.

## 2 Methods

### 2.1 Search strategy

**FIGURE 1:**
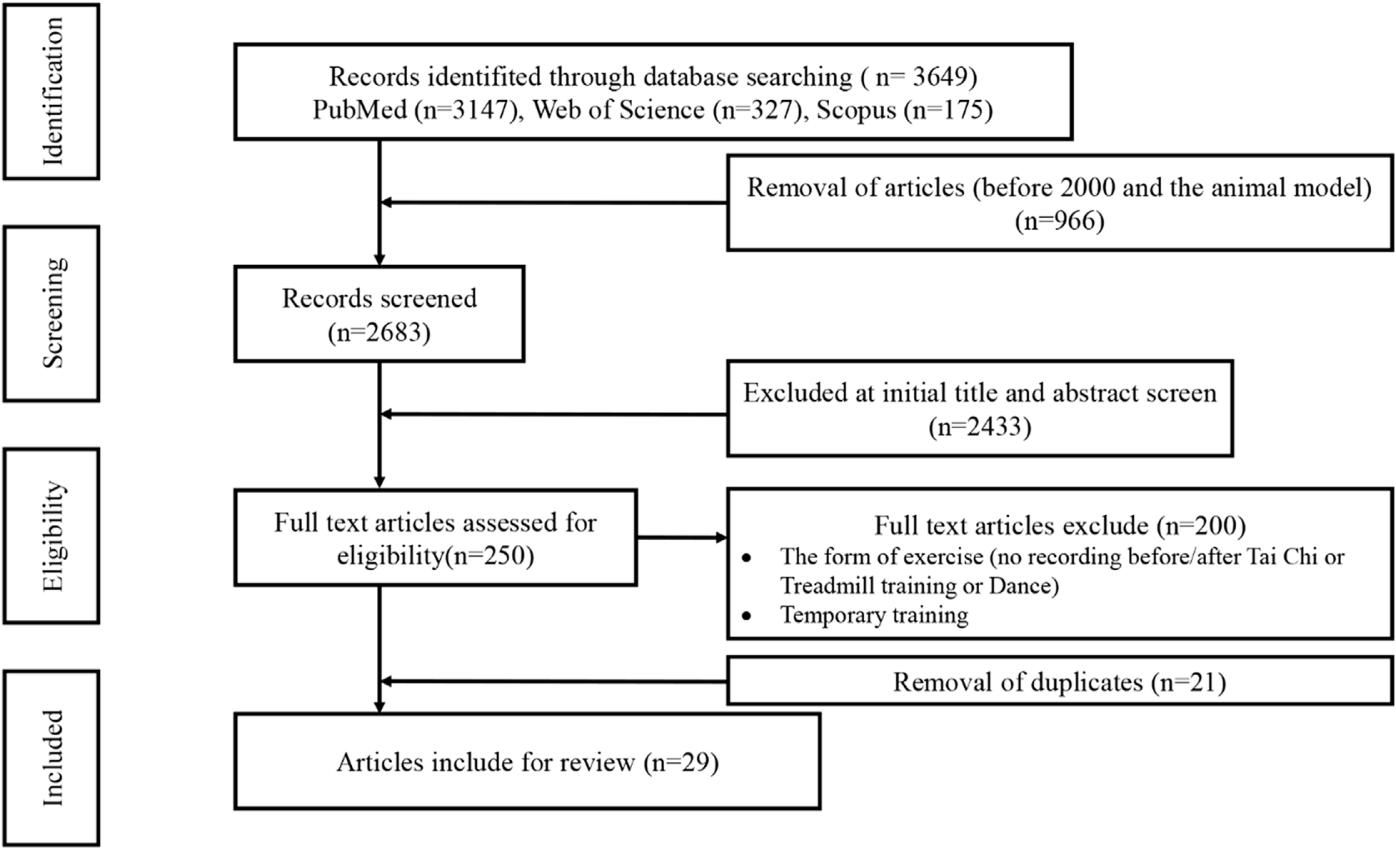
PRISMA flow chart with information at different stages of the study process.

The PubMed, Web of Science, and Scopus database were searched from January 2000 to October 2021 in this study. Then, the keywords, synonyms, search strategies, and Boolean logic operators were used for retrieval by the reviewer (LW), including four fields (connected with “AND”) with independent search terms (Table 1). The first search field focused on the participants, who were categorized according to the population of interest (i.e., older adults, healthy older adults, and older adults with PD). The second search field included possible synonyms for the forms of exercise, i.e., Tai Chi (Tai Chi Chuan), treadmill (walking) exercise, and dancing (dance). The third search field comprised synonyms for the brain function and included “brain,” “neural,” and “neuronal.” The fourth search field focused on the measurement device of interest to assess the cortical activity (i.e., EEG, fNIRS, and fMRI), these three kinds of detection devices are commonly used for detecting brain activity. All key search terms were matched and explored with medical subject headings (MeSH). Moreover, the reference lists of the included studies were also searched. The PRISMA flow chart of study design, which includes the search information at each stage of the study process, is presented (Figure 1).

**TABLE 1:** Key search words and synonyms used for each search field.

Population	Exercise	Brain activity	Measurement technique
TITLE-ABS-KEY	TITLE-ABS-KEY	TITLE-ABS-KEY	TITLE-ABS-KEY
“Older adult”	“Tai Chi”	“Brain”	“EEG”
“Healthy elder”	“Tai Chi Chuan”	“Neural”	“electroencephalography”
“Elderly”	“Treadmill (walking) exercise”	“Neuronal”	“fNIRS”
“Parkinson”	“Dancing”		“functional near-infra spectroscopy”
“Parkinson’s disease”	“Dance”		“fMRI”
			“functional magnetic resonance imaging”

### 2.2 Eligibility criteria

The inclusion and exclusion criteria were created by two reviewers (LW and FL). Studies were included if 1) it was printed in full English text; 2) the aim of the study was to examine the chronic effects of three terms of exercises on brain activity in healthy older adults and people with PD; 3) the target population: healthy older adults and people with PD with a mean age of 60 years or above; 4) the studies included at least distinct exercise interventions or one exercise intervention (Tai Chi, treadmill training, and dancing) with a no-exercise controlled intervention (NE) or other types compared in each trial; 5) the studies included at least one measurement device (EEG, fMRI, or fNIRS). The searches included in this review were restricted to exercise intervention trials, which were published from January 2000 to October 2021.

### 2.3 Quality assessment of studies

The methodological quality was independently assessed according to the physiotherapy evidence database (PEDro) scale (ranging from 0 to 10 points). To reduce the risk of bias in assessment, the work was performed by two reviewers (LW and LT). Any disagreements in scores between reviewers were judged after discussing with the third reviewer.

### 2.4 Data extraction

Data were extracted by the reviewer (LW), synthesized into table format, and confirmed by another reviewer (LT), according to the information strategy. The data extracted included authors, the year of publication, demographic, experimental protocol, measurement device, regions of interest, signal pre-processing, data outcomes, and conclusion.

## 3 Results

### 3.1 Study selection

The search strategy fielded 3,649 articles according to the key terms from three publication databases (Moher et al., 2009). Following the screening of the title and abstract and removal of duplicates, 29 articles were included according to the inclusion and exclusion criteria, 24 of which were finally included in the group of healthy older adults, and five of which were in the group of people with PD. Fourteen of the twenty-four reported Tai Chi (Fong et al., 2014; Tao et al., 2016; Tao et al., 2017a; Tao et al., 2017b; Liu et al., 2018; Port et al., 2018; Wu et al., 2018; Liu et al., 2019; Mei et al., 2019; Yang et al., 2019; Yue et al., 2020a; Yue et al., 2020b; Yue et al., 2020c; Liu et al., 2020), four studies investigated treadmill training (Smith et al., 2013; Chuang et al., 2015; Chirles et al., 2017; Won et al., 2021), and seven studies included dancing (Chuang et al., 2015; Eggenberger et al., 2016; Azman et al., 2017; Ji et al., 2018; Zilidou et al., 2018; Voss et al., 2019; Balazova et al., 2021). Five studies reported treadmill training in people with PD (Carvalho et al., 2015; Maidan et al., 2017; Maidan et al., 2018; Calabro et al., 2019; Droby et al., 2020). Articles on healthy older adults were more in number than those on patients with PD. Tables 2, 3 summarize the essential characteristics of these studies in healthy older adults and people with PD.

**TABLE 2:** Characteristics of the studies in healthy older adults.

Studies	Participants	Experimental protocol	Measurement device and outcome measures	Regions of interest (ROIs)	Signal pre-processing	Others
Tai Chi						
fNIRS						
Yang et al. (2019) (OA)	TCC:66.31 ± 4.25	TCC: 45 min per session for 3 days per week, 8 weeks;	fNIRS	Frontal_Sup_L Frontal_Inf_L Frontal_Sup_R Frontal_Inf_R	xTopo software for the pre-processing;	The Flanker task was performed during the assessment on neuroimaging
	CG:65.92 ± 3.48		the ETG-4000 system (10 Hz); 44channels;		Signals with frequencies of less than 0.04 Hz and more than 0.50 Hz were filtered out	
			Outcome measures: change in oxygenated haemoglobin			
EEG						
Fong et al. (2014) (OA)	YA:22.43 ± 2.58	TCC: 3 times a week and for 30 min per session, at least 5 years of exercise	EEG	Fz, Cz, Pz	Signals band-pass filtered with cut-off frequency of 0.1–50 Hz, a notch filter of 60 Hz to acquire data;	The task-switching task was completed as the stimulation to acquire the EEG data
	TCC:67.31 ± 4.92		Ag-AgCl electrodes (1000 Hz);	reference channel: right and left mastoid electrodes, Fpz;	segmented into 1200 ms epochs	
	OEE:68.37 ± 3.68		14 channels (Fz, F3, F4, Cz, C3, C4,			
	OSL:68.93 ± 4.28		Pz, P3, P4, T3, T4, Oz, O1, and O2);			
			Outcome measures: P3 amplitude and latency			
fMRI						
Yue et al. (2020a) (OA)	TCC:62.90 ± 2.40	TCC: 5 × 90-min sessions per week and more than 6 years	fMRI	HIP_L	RESTplus software for pre-processing, including: deletion of data, time layer, and head motion correction, the registration, removal of artefacts, and temporal band-pass filtering (0.01–0.08 Hz)	This study also assessed the global cognition by the MOCA (Montreal Cognitive Assessment Scale)
	WG:63.27 ± 3.60		3.0 T system;	PHG_L		
			Outcome measures: regional homogeneity (ReHo)	FG_L		
Tao et al. (2016) (OA)	TCC:62.38 ± 4.55	TCC: 5 days per week for 12 weeks with each session lasting 60 min	fMRI	The seeds:	The Statistical Parametric Mapping (SPM8) for pre-processing of fMRI data, including slice-timing, realignment, co-registration to structural image, normalization and smoothing, and band-pass filtering (0.01–0.1 Hz)	This study also performed the memory function measurement by the Wechsler Memory Scale-Chinese Revision (WMS-CR)
	BDJ:62.18 ± 3.79		3.0 T-GE scanner (eight-channel);	BHPC, mPFC		
	CG:59.76 ± 4.83		Outcome measures: the BOLD signal			
Port et al. (2018) (OA)	TCC:66.4 ± 4.9	TCC: 2 times per week, at least 3 years	fMRI	NR	The FSL software for data pre-processing, the step included movement correction, high-pass filter, spatial smoothing, and normalization	The N back task and SWCT were also performed while acquiring the fMRI data.
	WA:66.4 ± 7.0		3.0-T GE scanner (32 channels)			
			Outcome measures: the BOLD signal			
Yue et al. (2020b) (OA)	TCC:62.9 ± 2.38	TCC: 1.5 h per session, 5 sessions a week, more than 6 years	fMRI	DMN, SMN, VN	The imaging (fMRI) data were pre-processed by the DPABI 4.3 toolbox, including data format conversion, removal, slice timing, and head motion correction, spatial normalization, and smooth processing	NR
	WG:63.27 ± 3.58		3 T scanner			
			Outcome measures: the BOLD signal			
Liu et al. (2019) (OA)	TCC:62.38 ± 4.55	TCC: 5 days per week for 12 weeks with each session 60 min	fMRI	DMN;	The SPM 8 were used for the pre-processing, images were realigned, segmented, spatial normalization, Gaussian smoothing, band-pass filtering (0.008–0.09 Hz), and motion correction	This study also performed the memory function measurement by the WMS-CR
	BDJ:62.18 ± 3.79		3.0 T-GE scanner (eight-channel);	The seeds: PCC and mPFC		
	CG:59.76 ± 4.83		Outcome measures: the BOLD signal			
Tao et al. (2017a) (OA)	TCC:62.38 ± 4.55	TCC: a 60-min Tai Chi Chuan practice session 5 days per week for 12 weeks	fMRI	CNN	The DPARSF software for all pre-processing steps, including realignment, head-motion correction, normalization, segmentation, smoothing, and bandpass filtering (0.01–0.08 Hz)	The WMS-CR was performed in this study to evaluate the memory functions
	BDJ:62.33 ± 3.88		3.0 T GE scanner (eight-channel);			
	CG:59.76 ± 4.83		Outcome measures: the BOLD signal			
Tao et al. (2017b) (OA)	TCC:62.38 ± 4.55	TCC: 60 min per session, 5 days per week for 12 weeks	fMRI	DLPFC, mPFC	The DPARSF software for the fMRI data pre-processing, including slice timing correction, realignment, segmentation, head-motion correction, normalization, and smoothing	This study also assessed the memory functions by the WMS-CR
	BDJ:62.33 ± 3.88		3.0 T GE scanner (eight-channel);			
	CG:59.76 ± 4.83		Outcome measures: the BOLD signal, fALFF			
Wu et al. (2018) (OA)	TCC:64.9 ± 2.8	TCC: 60 min per session, three weekly sessions for 12 weeks	fMRI	PFC	The SPM 12 toolbox was used for image preprocessing, the step included excluding the 6-s dummy scan, slice timing correction, head motion correction, registration, normalization, and Gaussian filter	The Stroop task was performed during the fMRI scans
	CG:64.9 ± 3.2		3.0-T Trio MRI (32-channel);			
			Outcome measures: the BOLD signal			
Yue et al. (2020c) (OA)	TCC:62.9 ± 2.38	TCC: 90 min per week, 5 sessions a week, at least 6 years	fMRI	NR	The PANDA software for the data preprocessing, including head motion correction, removal, and calculating the fractional anisotropy of every voxel	The GRETNA toolbox was also used to calculate the topological attributes of brain functional networks in this study
	WG:63.27 ± 3.58		3.0 T MRI scanner (32 channel);			
			Outcome measures: the BOLD signal			
Mei et al. (2019) (OA)	TCC:62.81 ± 3.02	TCC: 90 min per session, 4–6 sessions a week, more than 6 years	fMRI	LFG	The DPABI software for the fMRI data pre-processing, this step included the removal, slice timing, and head-motion correction, spatial normalization, and Gaussian smoothing	The 2-back task also was performed to evaluate the cognitive function
	WG:63.55 ± 3.04		3.0-T GE (channel: NR)			
			Outcome measures: the BOLD signal, the fALFF			
Liu et al. (2020) (OA)	TCC:64.94 ± 2.37	TCC: an average of 9.98 ± 5.16 years	fMRI	VS., ACC	The SPM 12 toolbox for data preprocessing, including realignment, segmentation, image registration, slice timing, spatial normalization, and Gaussian smoothing	This study performed a sequential decision task, determined all participants’ total number of coins while undergoing fMRI scanning
	CG:64.06 ± 3.05		3 T Siemens Trio system (channel: NR)			
			Outcome measures: the BOLD signal			
Liu et al. (2018) (OA)	TCC:65.19 ± 2.30	TCC: 66.76 ± 20.51 min per day, more than 10 years	fMRI	DLPFC, and as the seed	Data pre-processing was performed using the DPARSFA software, the step included removal, delay correction, realignment, and head motion correction, segmentation, spatial normalization, Gaussian smoothing, and bandpass filter (0.01–0.08 Hz)	The Beck Depression Inventory, the NEO Five-Factor
	CG:63.92 ± 2.87		3.0-T Siemens Trio system scanner (channel: NR);			Inventory, the Five
			Outcome measures: the BOLD signal			Facets Mindfulness Questionnaire, and the Mindful Attention Awareness Scale were used before scanning, and a sequential decision task was undertaken after the fMRI scanning
Treadmill training						
EEG						
Chuang et al. (2015) (OA)	TT:67.01 ± 1.67	TT: 3 sessions of 30 min per week for 12 weeks;	EEG	The referenced electrode: earlobes, Fpz;	The Scan software for the ERP data, the step included replacement, signal correction, and the low-pass filter (30 Hz)	The Flanker task was performed while the EEG recording
	DDR:69.43 ± 3.82	The training intensity: 40–60% of HRmax and maintain 50% HRmax	NeuroScan NuAmps acquisition	The electrodes of interest: NR		
	CG:68.25 ± 3.96		Amplifiers (500 Hz);			
			The channel: NR;			
			Outcome measures: ERP (N2, P3)			
fMRI						
Chirles et al. (2017) (OA)	CN:76.1 ± 7.2	CN: 30 min per time, four times a week for 12 weeks;	fMRI	The seeds: PCC, precuneus	The Analysis of Functional NeuroImages (AFNI) software for the fMRI data, the step included alignment, slice time, and motion correction, bandpass filter (0.005–0.10 Hz), Gaussian smoothing, normalization	This study also performed the neuropsychological test battery including the Mini-Mental State Exam etc.
	MCI:79.6 ± 6.8	The training intensity: 50–60% of HRR (heart rate reserve)	General Electric 3.0 T scanner; the channel: NR;			
			Outcome measures: the BOLD signal			
Won et al. (2021) (OA)	CN:75.3 ± 7.4	CN: 30 min per session, 4 sessions a week for 12 weeks;	fMRI	The seeds: hippocampus	The AFNI software for data preprocessing, including removal, motion correction, alignment, and reduction of the physiological noise	Some tasks (i.e. the Rey Auditory Verbal
	MCI:78.8 ± 7.6	The training intensity: gradually increase and maintain 50–60% of HRR	3.0 T GE MRI scanner;			Memory Test etc.) were performed to evaluate the memory function in this study
			The channel: NR;			
			Outcome measures: the BOLD signal			
Smith et al. (2013) (OA)s	CN:76.0 ± 7.3	CN: 30 min per session, 4 sessions per week for 12 weeks;	fMRI	All voxels	The ANFI software for the fMRI image, the step included removal, shift, registration, alignment, Gaussian smoothing, and extract hemodynamic response functions (HRFs)	The famous name recognition task was performed while the fMRI scanning
	MCI:78.7 ± 7.5	The training intensity: gradually increase and maintain 50–60% of HRR	the General Electric 3.0 T scanner;			
			The channel: NR;			
			Outcome measures: the BOLD signal			
Dancing						
fNIRS						
Eggenberger et al. (2016) (OA)	DANCE:72.8 ± 5.9	DANCE: 30-min per session, 3 sessions per week for 8 weeks	fNIRS	PFC	The fNIRS data, from the two sensors on the left and right PFC, were pre-processed separately including the high-pass filter, minimized bias from Mayer waves, and visual inspection for motion artifacts	The treadmill walking protocol was applied during the fNIRS scanning
	BALANCE:77.8 ± 7.4		the Oxiplex Tissue Spectrometer system (1 Hz);			
			6 channels;			
			Outcome measures: HbO2 and Hb			
Azman et al. (2017) (OA)	CN and MCI: 73.6 ± 1.8	CN: 20-min per person, 2 times per week for 6 weeks each session, 2 sessions	fNIRS	FPC	The pre-processed step of fNIRS data including the low-pass filtered and averaged by the Savitzky–Golay filter, baseline correction, and normalization	The blood concentrations were measured while playing dance video game
			the BRAIN-NIRS hb13 system (2 Hz);			
			The channel: NR;			
			Outcome measures: HbO2 and Hb			
EEG						
Zilidou et al. (2018) (OA)	DANCE:68.73 ± 4.73	DANCE: 60 min 2 times per week for 24 weeks (at least 28 sessions)	EEG	All voxels	The preprocessing was performed by EEGLAB, the step included a high-pass filter (1 Hz), low-pass filter (100 Hz), band-stop filter (47–53Hz, 97–103Hz, 147–153 Hz), and remove source component (ICA)	The neuropsychological and physical assessments were performed at baseline and after the training intervention
	CN:66 ± 5.51		the Nihon			
			Kohden JE-207A device (500 Hz);			
			57 channels;			
			Outcome measures: functional connection and global network			
Chuang et al. (2015) (OA)	TT:67.01 ± 1.67	DDR: 3 sessions of 30 min per week for 12 weeks	EEG	The referenced electrode: earlobes, Fpz;	The Scan software for the ERP data, the step included replacement, signal correction, and the low-pass filter (30 Hz)	The Flanker task was performed while the EEG recording
	DDR:69.43 ± 3.82		NeuroScan NuAmps acquisition	The electrodes of interest: NR		
	CG:68.25 ± 3.96		Amplifiers (500 Hz);			
			The channel: NR;			
			Outcome measures: ERP (N2, P3)			
fMRI						
Ji et al. (2018) (OA)	DANCE:74.24 ± 6.29	DANCE: 45 min per time, 4 times a week for 6 weeks;	fMRI	Executive network, attentional network, salience network, memory	The fMRI data were pre-processed using the CONN toolbox including slice timing, realignment, segmentization, coregistration, normalization, and smoothing	During each neuroimaging session, the following image acquisition protocols were used, the task was a block-event mixed design modified from Zeineh’s paper
		The training intensity: maintain the 65%–75% HRmax	the Philips 3 Tesla (T) TX Achieva scanner (the channel: NR);	network, default-mode network, visual network, and motor network		
			Outcome measures: the BOLD signal			
Balazova et al. (2021) (OA)	DANCE:69.2 ± 5.47	DANCE: 60 min per lesson, 3 times a week for 6 months;	fMRI	Cerebellum, DMN, visual network, right and left	The fMRI data were pre-processed by the SPM 12 toolbox, pre-processing included realignment, unwarping, normalization, and spatial smoothing	The neuropsychological test was used to evaluate the global cognitive functions (memory, attention, language etc.)
	CN:69.0 ± 6.08	The training intensity: the medium intensity	3T Siemens Prisma MR scanner (the channel and sampling frequency: NR);	frontoparietal network, language network, salience (insulo-opercular) network, frontoparietal control network, and sensory-motor network		
			Outcome measures: the BOLD signal			
Voss et al. (2019) (OA)	CN:65.85 ± 4.29	DANCE: 3 times per week for 60 min each for 6 months;	fMRI	DMN, SAL, DAN, ECN	The fMRI data were pre-processed using tools from FSL, AFNI, FreeSurfer, the pre-processing including motion and distortion correction, extraction, spatial smoothing, temporal filtering (0.008–0.08 Hz), and nuisance regression	The cognitive assessment was completed including memory, vocabulary etc.
	DANCE:65.66 ± 4.62	The training intensity: NR	the 3.0 T Siemens Trio Tim system (the channel and sampling frequency: NR);			
	WG:65.49 ± 4.67		Outcome measures: the BOLD signal			
	WG+:64.62 ± 4.10					

OA, Older adult; TCC, Tai Chi Chuan; CG, Control group; YA, Young adult; OEE, Older adults performing endurance exercise; OSL, Older adults with a sedentary lifestyle; WG, Walking group; BDJ, Baduanjin; TT, Treadmill training; VR, Virtual reality; ST, Strength training; P, Physiotherapy; MF, Mean frequency; MMSE, the Mini-Mental State Examination; 10-MWT, the 10-m Walk Test; DDR, Dance dance revolution; ERP, Event-related potential; RAS, Rhythmic auditory stimulation; MCI, Mild cognitive impairment; WA, Water aerobics; LRGP, Low real gain percent-age; MRGP, Middle real gain percent-age; HRGP, High real gain percent-age; BALANCE, Balance and stretching training; SWCT, Stroop Word Color Task; fALFF, fractional amplitude of low-frequency fluctuations; CBSI, correlation–based signal improvement; NR, Not reported; WSM (LM), the Logical Memory subtest of the Wechsler Memory Scale. Cortical areas: HIP_L: Left hippocampus, PHG_L, Left parahippocampal gyrus, FG_L: Left fusiform, BHPC: Bilateral hippocampal, mPFC: medial prefrontal cortex, DMN: Default mode network, SMN: Sensory-motor network, VN: Visual network, PCC: Posterior cingulate cortex, CNN: Cognitive control network, DLPFC: Dorsolateral prefrontal cortex, PFC: Prefrontal cortex, L SFG: Left superior frontal gyrus, R MFG: Right middle frontal gyrus; L IFGt: Left inferior frontal gyrus pars triangularis; L MFG, Left middle frontal gyrus, VS, Ventral striatum, ACC: Anterior cingulate cortex; IFG, inferior frontal gyrus; MTG, Middle temporal gyrus; BA, Brodmann area; CBL, Cerebellar network; ECN, Executive control network, LMN, Lateral motor network; DAN, Dorsal attention network; FSN, Fronto-striatal network; BGN, Basal ganglia network; FPC, Frontopolar cortex; SAL, Salience network; LFG, Left frontal gyrus.

**TABLE 3:** Characteristics of the studies in people with PD.

Studies	Participants	Experimental protocol	Measurement device and outcome measures	Regions of interest (ROIs)	Signal pre-processing	Others
Tai Chi						
fNIRS						
Maidan et al. (2018) (PD)	TT:73.1 ± 1.1	TT: 45 min in each session, 3 sessions per week for 6 weeks;	fNIRS	PFC	The bandpass filter (0.01–0.14) to reduce physiological noise and signal draft, a wavelet filter to remove motion artefacts, CBSI	The assessment protocol included the general physical and cognitive performance was performed at pre-and post-training, and the gait and prefrontal activation were assessed during 3 walking tasks
	TT + VR:70.1 ± 1.3	The training intensity: NR	the PortaLite fNIRS system (10 Hz);			
			6 channels;			
			Outcome measures: oxygenated hemoglobin (HbO2)			
EEG						
Carvalho et al. (2015) (PD)	TT:64.8 ± 11.9	TT: 30 min per session, twice a week for 12 weeks;	EEG	Frontal pole, Frontal, Central, Temporal,	NR	Some tests (i.e., 10-m Walk Test, Unified Parkinson’s Disease Rating Scale-III etc.)
	ST:64.1 ± 9.9	The training intensity: 60% of the maximum oxygen consumption (VO2max) or 70% of the maximum	the system and channel: NR;	Parietal,		
	P:62.1 ± 11.7	Heart rate (HRmax)	Outcome measures: mean frequency (MF)	and Occipital		
Calabro et al. (2019) (PD)	TT:73 ± 8	TT: 30 min one time, 5 times per week for 8 weeks;	EEG	The frontal, centroparietal, temporal areas	The band-pass filter (1–200 Hz) using a zero-phase finite impulse response (FIR) to filter minimize drifts and notch-filtered at 50 Hz to remove the power-line noise during sampling	EEG data was acquired while the walking;
	TT + RAS:70 ± 8	The training intensity: NR	Brain-Quick system (512 Hz);		The EEGLab software for data pre-processing, including re-bandpass filter (8–40 Hz), Independent Component	The time-frequency coherence was also computed by the EEG data
			The channel: 19;		Analysis (ICA)	
			Outcome measures: α (8–12 Hz), β (13–28 Hz) frequency			
fMRI						
Maidan et al. (2017) (PD)	TT:71.5 ± 1.5	TT: 45 min per time, 3 times per week for 6 weeks;	fMRI	IFG, the left cerebellum anterior lobe, MTG, the right BA 10	The fMRI data were pre-processed using SPM 12 software	The imaged walking fMRI task were projected to the participants in the MRI scanner
	TT + VR:71.2 ± 1.7	The training intensity: NR	the 3 T GE scanner (8-channel);			
			Outcome measures: the BOLD signal			
Droby et al. (2020) (PD)	TT:73.6 ± 6.5	TT: 45 min per session, 3 sessions/week for 6 weeks;	fMRI	CBL, ECN, LMN, SMN, DAN, FSN, BGN	The fMRI data were pre-processed, the step included slice time correction, realignment, head-motion correction, normalization, and smoothing	The cognitive, gait and balance assessments were performed at baseline and after motor training
	TT + VR:72.8 ± 6.7	The training intensity: NR	3 T Signa Excite MR scanner (8-channel);			
			Outcome measures: the BOLD signal			

OA: Older adult, TCC: Tai Chi Chuan, CG: Control group, YA: Young adult, OEE: Older adults performing endurance exercise, OSL: Older adults with a sedentary lifestyle, WG: Walking group, BDJ: Baduanjin, TT: Treadmill training, VR: Virtual reality, ST: Strength training, P: Physiotherapy, MF: Mean frequency, MMSE: the Mini-Mental State Examination, 10-MWT: the 10-m Walk Test, DDR: Dance dance revolution, ERP: Event-related potential, RAS: Rhythmic auditory stimulation, MCI: Mild cognitive impairment, WA: Water aerobics, LRGP: Low real gain percent-age, MRGP: Middle real gain percent-age, HRGP: High real gain percent-age, BALANCE: Balance and stretching training, SWCT: Stroop Word Color Task, fALFF: fractional amplitude of low-frequency fluctuations, CBSI: correlation–based signal improvement, NR: Not reported, WSM (LM): the Logical Memory subtest of the Wechsler Memory Scale. Cortical areas: HIP_L: Left hippocampus, PHG_L: Left parahippocampal gyrus, FG_L: Left fusiform, BHPC: Bilateral hippocampal, mPFC: medial prefrontal cortex, DMN: Default mode network, SMN: Sensory-motor network, VN: Visual network, PCC: Posterior cingulate cortex, CNN: Cognitive control network, DLPFC: Dorsolateral prefrontal cortex, PFC: Prefrontal cortex, L SFG: Left superior frontal gyrus, R MFG: Right middle frontal gyrus, L IFGt: Left inferior frontal gyrus pars triangularis, L MFG: Left middle frontal gyrus, VS.: Ventral striatum, ACC: Anterior cingulate cortex, IFG: inferior frontal gyrus, MTG: Middle temporal gyrus, BA: Brodmann area, CBL: Cerebellar network, ECN: Executive control network, LMN: Lateral motor network, DAN: Dorsal attention network, FSN: Fronto-striatal network, BGN: Basal ganglia network, FPC: Frontopolar cortex, SAL: Salience network, LFG: Left frontal gyrus.

### 3.2 Characteristics of included studies

The participants included healthy older adults and patients with PD with average ages of 69.7 ± 6.3 and 65.3 ± 4.2 years of age in the selected articles, respectively. The range of disease severity of PD was assessed by using the Hoehn and Yahr scale (Hoehn and Yahr, 2001), from 1 to 2 (mild to moderate level).

The duration and frequency of Tai Chi intervention ranged from 6 weeks to 10 years, 2–7 sessions/week, and 30–90 min/session; the duration and frequency in treadmill training included 12 weeks, 3–4 sessions/week, and 30 min/session; the training intensity gradually increased and was maintained at 50–60% of heart rate reserve (HRR); the duration and frequency of dancing intervention ranged from 6 to 24 weeks, 2–4 sessions/week, and 20–60 min/session in healthy older adults, while the duration and frequency of treadmill training ranged from 6 to 12 weeks, 2–5 sessions/week, 30–45 min/session in people with PD.

Of the 29 studies, 14 studies reported that brain activity was assessed by use of different types of tasks (e.g., N-back task, Flanker task, walking, etc.) while participants were acquiring data pre- and the post-training, namely, 11 studies in healthy older adults (Smith et al., 2013; Fong et al., 2014; Chuang et al., 2015; Eggenberger et al., 2016; Azman et al., 2017; Ji et al., 2018; Port et al., 2018; Wu et al., 2018; Mei et al., 2019; Yang et al., 2019; Liu et al., 2020) and three studies in people with PD (Maidan et al., 2017; Maidan et al., 2018; Calabro et al., 2019). On the other hand, many regions of interest such as prefrontal cortex (PFC), anterior cingulate cortex (ACC), and bilateral hippocampal (BHPC) were included in healthy older adults and people with PD.

### 3.3 Methodological quality

The scores for each criterion using the PEDro scale are presented in Table 4. The mean score for all 29 trials was 5.52 ± 1.33. Across the 29 studies, neither the participants nor the therapists administering the program were blinded to the intervention. Only two studies, which were separately derived from the group of healthy older adults and people with PD, concealed the allocation of all participants, reported blinded assessors, and used intention-to-treat analysis (Maidan et al., 2018; Wu et al., 2018). Several studies recruited and allocated participants based on exercise-related experiences, and thus failed to meet the requirement of random allocation (Fong et al., 2014; Liu et al., 2018; Port et al., 2018; Mei et al., 2019; Yue et al., 2020a; Yue et al., 2020b; Yue et al., 2020c; Liu et al., 2020), leading to the lower score.

**TABLE 4:** PEDro scale of quality for eligible trials.

Study	EC	RA	CA	SAB	SB	TB	AB	DR	ITA	BC	PM	OSQ
Yang et al. (2019)	YES	1	1	1	0	0	1	1	0	1	1	7
Fong et al. (2014)	YES	0	0	0	0	0	0	1	1	1	1	4
Yue et al. (2020a)	YES	0	0	0	0	0	1	1	0	1	1	4
Tao et al. (2016)	YES	1	1	1	0	0	1	0	0	1	1	6
Port et al. (2018)	YES	0	0	1	0	0	1	1	1	1	1	6
Yue et al. (2020b)	YES	0	0	0	0	0	1	1	0	1	1	4
Liu et al. (2019)	YES	1	1	1	0	0	1	0	0	1	1	6
Tao et al. (2017a)	YES	1	1	1	0	0	1	0	0	1	1	6
Tao et al. (2017b)	YES	1	1	1	0	0	1	0	0	1	1	6
Wu et al. (2018)	YES	1	1	1	0	0	1	1	1	1	1	8
Yue et al. (2020c)	YES	0	0	0	0	0	1	1	0	1	1	4
Mei et al. (2019)	YES	0	0	0	0	0	1	1	0	1	1	4
Liu et al. (2020)	YES	0	0	0	0	0	1	1	0	1	1	4
Liu et al. (2018)	YES	0	0	0	0	0	1	1	0	1	1	4
Maidan et al. (2018)	YES	1	1	1	0	0	1	1	1	1	1	8
Carvalho et al. (2015)	YES	1	1	1	0	0	0	1	1	1	1	7
Chuang et al. (2015)	YES	1	0	1	0	0	0	1	0	1	1	5
Calabro et al. (2019)	YES	1	0	1	0	0	1	1	1	1	1	7
Chirles et al. (2017)	YES	0	0	0	0	0	1	1	0	1	1	4
Won et al. (2021)	YES	0	0	0	0	0	1	1	0	1	1	4
Smith et al. (2013)	YES	0	0	0	0	0	1	1	0	1	1	4
Maidan et al. (2017)	YES	1	0	1	0	0	1	1	1	1	1	7
Droby et al. (2020)	YES	1	0	1	0	0	0	1	0	1	1	5
Eggenberger et al. (2016)	YES	1	1	1	1	0	0	1	0	1	1	7
Azman et al. (2017)	NO	0	0	1	0	0	0	1	1	1	1	5
Zilidou et al. (2018)	NO	1	0	1	0	0	0	1	0	1	1	5
Ji et al. (2018)	YES	1	0	1	0	0	0	1	1	1	1	6
Balazova et al. (2021)	YES	1	1	1	0	0	0	1	0	1	1	6
Voss et al. (2019)	YES	1	0	1	0	0	1	1	1	1	1	7

EC, eligibility criteria; RA, random allocation; CA, concealed allocation; SAB, similar at baseline; SB, subject blinded; TB, therapist blinded; AB, assessor blinded; DR, drop-out rate; ITA, intention-to-treat analysis; BC, between-group comparison; PM, point measures; OSQ, overall study quality.

### 3.4 Key findings of healthy older adults

The key findings and conclusions of the studies are reported in Table 5. In Tai Chi group, many benefits in cognitive function, memory, and emotion were observed after healthy older adults receiving different types of exercise interventions. Increased activation during the task (i.e., switch task and flanker task) were found in PFC, left superior frontal, bilateral cerebellum, and right posterior cingulate cortex after Tai Chi practice, suggesting that it can improve inhibitory control ability and emotion (Wu et al., 2018; Yang et al., 2019; Liu et al., 2020). Similarly, changes in regional homogeneity (ReHo), amplitude of low-frequency fluctuations (fALFF) were observed in the left medial temporal lobe, para hippocampus, left fusiform gyrus, and dorsolateral prefrontal cortex (DLPFC), and it was related to improvement of memory (Tao et al., 2017b; Mei et al., 2019; Yue et al., 2020a). Increased functional connectivity between the bilateral hippocampus and medial PFC substantiated this result (Tao et al., 2016). However, some studies reported that the functional connectivity between DLPFC and left superior frontal gyrus, DLPFC and middle frontal gyrus was decreased, implying that a negative relationship between emotion regulation and functional connectivity (Tao et al., 2017a; Liu et al., 2018). Both studies showed that Tai Chi contributed to enhanced brain activity in correlation to improved memory, emotion, and cognitive functions.

**TABLE 5:** Key findings and conclusions of the studies in healthy older adults.

Studies	Key findings	Conclusions
fNIRS		
Yang et al. (2019) (OA)	The Frontal_Sup_L, Frontal_Inf_L oxy-Hb signal in the TCC group was higher at post-test than at pre-test for the incongruent flanker task.	TCC has a positive impact to improve the inhibitory control in older adults
	The Frontal_Sup_L oxy-Hb signal in the TCC group was increased during the flanker task compared to the CG	
EEG		
Fong et al. (2014) (OA)	The P3 amplitude in the YA, OEE, and OTC groups was higher than the OSL group and no differences were observed between YA, OEE, and OTC	The endurance exercise and TCC may have beneficial effects on the cognitive function of older adults
fMRI		
Yue et al. (2020a) (OA)	The left medial temporal lobe, the hippocampus, and	Tai Chi exercise may improve the function of the hippocampus to enhance the memory performance in older adults
	para hippocampus ReHo signal in the TCG group were higher than in the WG group	
Tao et al. (2016) (OA)	Increased functional connectivity between the	Tai Chi may be effective in preventing memory decline during aging
	bilateral hippocampus and right mPFC and left mPFC was observed in TCC group compared to the CG	
Port et al. (2018) (OA)	The right intra calcarine cortex,	Tai Chi exercise may have a positive effect to improve the cognitive performance in older adults
	lateral occipital cortex, and occipital pole BOLD response in the TC group was lower than the WA group during the SWCT;	
	Less BOLD response in the right superior frontal gyrus and frontal pole was presented in the TC group compared to the WA group during N-back task	
Yue et al. (2020b) (OA)	There were significant differences in DMN, SMN, and VN between the TCC group and WG	Long-term exercise (Tai Chi or walking) has different impacts on the brain’s functional networks and brain functional plasticity of older adults
Liu et al. (2019) (OA)	Increased rsFC between the mPFC and right putamen/caudate was observed in the TCC group compared to the Baduanjin group;	Tai Chi exercise can modulate the DMN in older adults
	The rsFC between the PCC and the right putamen/caudate was increased in the TCC group compared to the CG	
Tao et al. (2017a) (OA)	Decreased rsFC between the DLPFC and the left SFG and ACC was observed in the TCC group compared to the CG	Tai Chi exercise has the significant potential to prevent cognitive function decline
Tao et al. (2017b) (OA)	Increased fALFF in the DLPFC in the slow-5 and low-frequency bands was observed in the TCC group compared to the CG;	Tai Chi may prevent memory decline during aging by certain mechanisms
Wu et al. (2018) (OA)	Increased left superior frontal activation for Switch > Non-switch was observed in the post-intervention compared to the pre-intervention;	TCC training may enhance the function of the prefrontal activation to improve the ability of task-switching
	Greater prefrontal activation in the switch condition was related to the greater reductions in task-switching errors	
Yue et al. (2020c) (OA)	The higher small-world attributes were observed in the TC group compared with the WG;	Tai Chi training is more useful to optimize the brain function and network of older adults
	The aggregation coefficient and local efficiency attributes were higher for the TC group than for the WG.	
Mei et al. (2019) (OA)	Increased fALFF in the LFG in the 0.01–0.08 Hz of the frequency channel was observed in the TCC group compared with the WG;	TCC exercise may prevent the decline of the cognitive function by improving the working memory in older adults
	Increased fALFF was related to the response time in the 2-back task	
Liu et al. (2020) (OA)	Increased bilateral cerebellum and right PCC activation was showed only in the Tai Chi group;	The long-term exercise may alleviate feelings of regret by strengthening the frontal functional connectivity
	The left with MFG, SFG, and IFG functional connectivity was higher at the poor outcome than the optimal outcome in the TC group	
Liu et al. (2018) (OA)	The rs-functional connectivity (rs-FC) between DLPFC and MFG was weaker in the TC group than the CG;	The long-term Tai Chi training can modulate non-judgment of inner experience on the emotion regulation of the older adults by decreasing functional
	This functional connectivity fully mediated the impact of non-judgment of inner experience in the TC group.	connectivity within the executive control network.
EEG		
Chuang et al. (2015) (OA)	The N2 and P3 latency were shortened in the TT group compared to the CG	Treadmill training can improve the cognitive function in older adults
fMRI		
Chirles et al. (2017) (OA)	Increased connectivity between the frontal and parietal regions was showed from before to after the intervention in healthy older adults	The treadmill training may be realized through the enhancement of neural recruitment mechanisms, to increase the cognitive reserve for the CG
Won et al. (2021) (OA)	Increased functional connectivity was observed between the posterior hippocampi and regions within the left cuneus and left precuneus after the intervention compared to before the intervention for healthy older adults	The hippocampal functional connectivity is increased by treadmill training to enhance the memory capacity for the CG
Smith et al. (2013) (OA)	Reduced activation during the task, is related to the semantic memory, was observed at the post- compared to the pre-intervention in the older adult	Treadmill exercise may improve neural efficiency to lead to the enhancement of cognitive function
fNIRS		
Eggenberger et al. (2016) (OA)	Reduced oxygenation during the acceleration of walking in the left and right hemispheric PFC was observed after the training;	Exercise training induced the change of PFC oxygenation correlated with the executive functions to improve the mobility and falls prevention in older adults
	The reduction of left-PFC oxygenation in the DANCE group was larger than the BALANCE group at the end of the 30-s walking task	
Azman et al. (2017) (OA)	Changes in the AUC (under the curve) value of the left and right PFC was different after the experiment	Dance training can be helpful in improving the cognitive function to enhance the motor-control ability
EEG		
Zilidou et al. (2018) (OA)	The dance training improved the optimal network performance as estimated by the small-world property;	Dance training can promote the mental and physical wellbeing of older adults as a non-pharmacological intervention
	The local network changes resulting in better information flow.	
Chuang et al. (2015) (OA)	Shortened N2 and P3 latencies were observed in the DDR group compared to the CG.	DDR training has a positive impact on the inhibitory control for older adults
fMRI		
Ji et al. (2018) (OA)	The involvement during the memory task in the motor cortices and cerebellum was greater at the post- than the pre-training;	Physical exercise can improve the gait speed and cognitive function by increasing involvement of motor-related networks
	Increased WMS (LM) was related to the motor network activation after the exercise.	
Balazova et al. (2021) (OA)	Increased rs-FC in the default mode network (DMN) and specific inter-networks were observed in the DANCE group compared to the CG, including insulo-opercular and right frontoparietal/frontoparietal control networks, visual and language/DMN networks, etc.	Dance training enhances the brain plasticity to improve the cognitive function
Voss et al. (2019) (OA)	No change on the functional connectivity of some networks at the post-training compared to the pre- in the DANCE group	NR

OA: Older adult, TCC: Tai Chi Chuan, CG: Control group, YA: Young adult, OEE: Older adults performing endurance exercise, OSL: Older adults with a sedentary lifestyle, WG: Walking group, BDJ: Baduanjin, TT: Treadmill training, VR: Virtual reality, ST: Strength training, P: Physiotherapy, MF: Mean frequency, MMSE: the Mini-Mental State Examination, 10-MWT: the 10-m Walk Test, DDR: Dance dance revolution, ERP: Event-related potential, RAS: Rhythmic auditory stimulation, MCI: Mild cognitive impairment, WA: Water aerobics, LRGP: Low real gain percent-age, MRGP: Middle real gain percent-age, HRGP: High real gain percent-age, BALANCE: Balance and stretching training, SWCT: Stroop Word Color Task, fALFF: fractional amplitude of low-frequency fluctuations, CBSI: correlation–based signal improvement, NR: Not reported, WSM (LM): the Logical Memory subtest of the Wechsler Memory Scale. Cortical areas: HIP_L: Left hippocampus, PHG_L: Left parahippocampal gyrus, FG_L: Left fusiform, BHPC: Bilateral hippocampal, mPFC: medial prefrontal cortex, DMN: Default mode network, SMN: Sensory-motor network, VN: Visual network, PCC: Posterior cingulate cortex, CNN: Cognitive control network, DLPFC: Dorsolateral prefrontal cortex, PFC: Prefrontal cortex, L SFG: Left superior frontal gyrus, R MFG: Right middle frontal gyrus, L IFGt: Left inferior frontal gyrus pars triangularis, L MFG: Left middle frontal gyrus, VS.: Ventral striatum, ACC: Anterior cingulate cortex, IFG: inferior frontal gyrus, MTG: Middle temporal gyrus, BA: Brodmann area, CBL: Cerebellar network, ECN: Executive control network, LMN: Lateral motor network, DAN: Dorsal attention network, FSN: Fronto-striatal network, BGN: Basal ganglia network, FPC: Frontopolar cortex, SAL: Salience network, LFG: Left frontal gyrus.

Four studies examined the change of brain activity in healthy older adults throughout the treadmill exercise from several sides. The brain electric activity—event-related potential (ERP) including N2 and P3, were shortened on the latency after exercise intervention (Chuang et al., 2015). Furthermore, one study identified an increase in the functional connectivity between the frontal and parietal regions, the posterior hippocampi, and regions within the left cuneus and left precuneus, whereas reduced activation which is related to semantic memory was observed in another study (Chirles et al., 2017; Won et al., 2021). The results of the three studies provided the evidence of treadmill exercise-induced benefits in improving cognitive performance and memory.

The effects of dancing exercise on brain activity in healthy older adults were showed in six studies. The functional connectivity between brain regions and the latency of N2 and P3 were utilized to assess the brain activity for healthy older adults after dancing training. The findings revealed that N2 and P3 latency were shortened (Smith et al., 2013) and the functional connectivity between insulo-opercular and right frontoparietal control networks, visual and language/DMN networks were increased, confirming the benefits of dancing exercise in the improvement of cognitive function (Chuang et al., 2015; Balazova et al., 2021). Eggenberger et al. (2016) investigated the oxygenation of older adults during walking. The participants showed reduced oxygenation during the acceleration of walking in PFC throughout the training. This study suggested that the exercise training induced the change of PFC oxygenation correlated with the executive functions to improve the mobility and help in prevention. Ji et al. (2018) analyzed the involvement of the brain region during memory tasks by using the fMRI data, and it observed that the involvement in motor cortices and the cerebellum during the task was increased during post-training. This study found that the dancing exercise can improve gait speed and cognitive function by increasing the involvement of motor-related networks.

### 3.5 Key findings of people with PD

Table 6 summarizes the key findings and conclusions of each study about people with PD. In the treadmill-training group, motor ability is the core index that researchers care about. Maidan et al. (2018) investigated the change in cortex activation during walking after treadmill practice and found a reduced activation in the prefrontal area compared to the baseline, suggesting that the pattern of cortex activation in PFC can be altered by treadmill training to reduce fall risk. The mean frequency (MF) and α, β belonged to the brain’s electric activity were used to study effects of exercise intervention on the brain activity in people with PD. Two studies showed that MF was higher in the left cerebral area and significant changes in α, β within the frontal and centroparietal electrodes were also observed, which might be associated with the improvement of physical ability and gait (Carvalho et al., 2015; Calabro et al., 2019). Additionally, increased activation in Brodmann area 10, inferior frontal gyrus, and functional connectivity in executive control network were observed after training, whereas the activation in the left cerebellum, middle temporal gyrus, and the functional connectivity in sensory-motor network (SMN) were decreased (Maidan et al., 2017; Droby et al., 2020). The findings of these two studies suggested that treadmill exercise may reduce the reliance on the frontal region and enhance the neural plasticity to improve gait and reduce falls. Enhanced brain activity is the evidence of improvement of motor ability throughout treadmill training.

**TABLE 6:** Key findings and conclusions of the studies in people with PD.

Studies	Key findings	Conclusions
fNIRS		
Maidan et al. (2018) (PD)	Reduced prefrontal activation during walking in PD was observed after the treadmill training compared to the baseline;	Prefrontal activation during usual and complex walking conditions can be altered by treadmill exercise to reduce fall risk
	The changes of activation in the left prefrontal cortex were larger than the right prefrontal cortex	
EEG		
Carvalho et al. (2015) (PD)	The MF in the TT group was higher than the P group, which is related to increased cortical activation;	Treadmill training in patients with PD can improve their physical ability and alleviate disease symptoms
	The activity area in the TT group was specific to the left cerebral compared with the other groups.	
Calabro et al. (2019) (PD)	Significant changes in gait-related α and β of ERS and ERD within the frontal and centroparietal electrodes were observed at post-training compared to the pre-training in the TT group;	Treadmill training may change the connectivity of frontocentroparietal/temporal to improve the gait
	Increased frontocentroparietal/temporal electrode connectivity was observed after the training in the TT group.	
fMRI		
Maidan et al. (2017) (PD)	Increased activation in the right BA 10 and IFG was observed after the training in the CG;	Treadmill training may reduce the reliance on the frontal region, leading to reduce falls and fall risk
	in contrast, decreased activation in the left cerebellum and MTG was showed after the training in this group	
Droby et al. (2020) (PD)	Decreased functional connectivity in BGN, ECN, and FSN was observed, and the functional connectivity in SMN was increased at the post-training in the CG;	Treadmill training may affect the neural pathways and enhance the neural plasticity to improve gait in PD
	these functional connectivity alterations were associated with improved usual and dual-task walking performance	

OA: Older adult, TCC: Tai Chi Chuan, CG: Control group, YA: Young adult, OEE: Older adults performing endurance exercise, OSL: Older adults with a sedentary lifestyle, WG: Walking group, BDJ: Baduanjin, TT: Treadmill training, VR: Virtual reality, ST: Strength training, P: Physiotherapy, MF: Mean frequency, MMSE: the Mini-Mental State Examination, 10-MWT: the 10-m Walk Test, DDR: Dance dance revolution, ERP: Event-related potential, RAS: Rhythmic auditory stimulation, MCI: Mild cognitive impairment, WA: Water aerobics, LRGP: Low real gain percent-age, MRGP: Middle real gain percent-age, HRGP: High real gain percent-age, BALANCE: Balance and stretching training, SWCT: Stroop Word Color Task, fALFF: fractional amplitude of low-frequency fluctuations, CBSI: correlation–based signal improvement, NR: Not reported, WSM (LM): the Logical Memory subtest of the Wechsler Memory Scale. Cortical areas: HIP_L: Left hippocampus, PHG_L: Left parahippocampal gyrus, FG_L: Left fusiform, BHPC: Bilateral hippocampal, mPFC: medial prefrontal cortex, DMN: Default mode network, SMN: Sensory-motor network, VN: Visual network, PCC: Posterior cingulate cortex, CNN: Cognitive control network, DLPFC: Dorsolateral prefrontal cortex, PFC: Prefrontal cortex, L SFG: Left superior frontal gyrus, R MFG: Right middle frontal gyrus, L IFGt: Left inferior frontal gyrus pars triangularis, L MFG: Left middle frontal gyrus, VS.: Ventral striatum, ACC: Anterior cingulate cortex, IFG: inferior frontal gyrus, MTG: Middle temporal gyrus, BA: Brodmann area, CBL: Cerebellar network, ECN: Executive control network, LMN: Lateral motor network, DAN: Dorsal attention network, FSN: Fronto-striatal network, BGN: Basal ganglia network, FPC: Frontopolar cortex, SAL: Salience network, LFG: Left frontal gyrus.

## 4 Discussion

To the best of our knowledge, this study is the first to cohesively present the effects of three training modalities on brain activity of healthy older adults and people with PD. Our systematic review of the literature found limited robust evidence for the effects of exercise on the brain activity of older adults and people with PD. Of all the trials included in our review, the objectives, and protocols of study are not comprehensive enough to definitely compare the differences between healthy older adults and people with PD after treadmill-training intervention. Therefore, while our systematic review showed that the improvement of cognitive function, memory, and emotion might be associated with modified brain activity, functional connectivity in healthy older adults through Tai Chi, treadmill training, and dancing, and revealed that treadmill exercise may improve the motor ability of people with PD by changing brain activity, it remained largely unknown if the difference was true.

In the group of healthy older adults, most studies included all exercise interventions (Tai Chi, treadmill training, and dancing) found that exercise can affect brain activity and functional connectivity to improve cognitive function, memory, or emotion, despite variability in types and dosages of the intervention used. An explanation for this could derive from their respective objectives and study protocols, 11 of the 24 studies utilized the cognitive or memory tasks in the experiment (such as N-back, flanker task, etc.), especially two of which were included in the treadmill exercise group (Smith et al., 2013; Chuang et al., 2015). Motor tasks, however, have yet to be incorporated into the study of the exercise intervention in healthy older adults.

In comparison to cognitive task in the healthy older adults group, of the five trials in group of people with PD, there were three trials including the motor task during the data collecting (i.e., walking, imaged walking) (Maidan et al., 2017; Maidan et al., 2018; Calabro et al., 2019). Therefore, we speculated that inconsistencies between oddball paradigms might lead to different performances between healthy older adults and patients with PD throughout treadmill training.

Only four studies from the group of healthy older adults and people with PD did not use the oddball paradigm during the data recording through treadmill training. Droby et al. (2020) found that an inverse change trend (such as increased or decreased) in functional connectivity between different brain regions was observed in people with PD after treadmill practice. This finding is consistent with previous studies which reported reduced hemispheric asymmetry in movement patterns due to age-related deficits in neural connectivity (Cabeza, 2002; Przybyla et al., 2011). Interestingly, there were two studies in healthy older adults suggesting an increased functional connectivity between some regions at post-training (Chirles et al., 2017; Won et al., 2021). The difference in change of functional connectivity was observed between three studies. To provide some explanations, three trials did not differ in the baseline demographic characteristics including age, education, sex, and the number of participants in the expected type of population. Three studies adopted the comparison between pre- and post-training within the treadmill training group or the healthy older adults group. Furthermore, the participants in a study by Chirles and Won underwent a 12-week treadmill training intervention including four sessions per week, while the duration of study by Droby was half of that amount of time including three sessions per week. Therefore, the inconsistency in the type of population and the intervention duration provided a sound reason for the disparate results.

In light of our findings about people with PD, we speculated the following. The study reported that cortical activity abnormally increases during walking in people with PD, and it might reflect a cortical compensation phenomenon (Stuart et al., 2018). Decreased cortical activation after training suggests that gait training improves the automaticity of walking and provides more stimuli, which in turn lowers the reliance on cognitive resources and a reflection of better utilization for motor networks during walking or during a task in patients with PD. This was similar to that found in people with PD (Maidan et al., 2016). As indicated from animal and human models, exercise can, to some degree, enhance neuroplasticity that promotes angiogenesis (growth of new blood vessels), neurogenesis (new functional neurons), and synaptogenesis (new synapses). All the processes are consistent with our findings, which reported that functional connectivity was increased (Bherer et al., 2013). Additionally, externally guided rhythmic movements may also account for the augmentation of these changes in PD participants (Rochester et al., 2005; Yogev et al., 2005; Herman et al., 2007; Maidan et al., 2017). Therefore, treadmill training might provide an external setting conducive to improving the gait in PD.

The use of measurement devices included fMRI and EEG presents a limitation in the current exercise intervention literature studies. In comparison to the fNIRS detection which allows moderate physical activity, fMRI and EEG require participants to possibly maintain a resting state. Even small motions may produce artifacts and noise during scanning (Power et al., 2012; Satterthwaite et al., 2012; Lin et al., 2020), affecting the final data analysis and results. Furthermore, most studies utilized the EEG and fMRI and adopted some tasks related to cognitive, memory, and inhibitory control. The type of task might be related to the limitation of EEG or fMRI. To explore the motor ability-related knowledge in people with PD, more feasible motor tasks should be developed and employed for fNIRS scanning.

The average score of PEDro in this review was 5.52, which suggested that the studies included were of moderate methodological quality (Moseley et al., 2002). A lack of some factors included random allocation, double blinding, and intention-to-treat analysis can be regarded as limitations of the methodology of all trials in this review. Furthermore, non-English studies, MRI-related articles were excluded and only three kinds of exercises were included here, which illustrate other limitations in this review. Additionally, only five of the studies and one exercise intervention (treadmill training) included targeted people with PD. Resultantly, our findings may not be representative in people with PD. There is a need for more robust and comprehensive studies on the exercise intervention for people with PD. For example, motor tasks are employed into the study of healthy older adults throughout the exercise, and more studies of people with PD adopt cognitive tasks during fNIRS, EEG, and fMRI scanning.

## 5 Conclusion

Our systematic review demonstrated that three terms of exercises (Tai Chi, treadmill training, and dancing) can modify brain activity, functional connectivity to improve cognitive function, memory, and emotion in healthy older adults, and treadmill exercise can improve the motor ability of people with PD, which was related to changes in the brain activity. However, with the current available studies, the differences of brain activity and performance between healthy older adults and patients with PD cannot be sufficiently confirmed in this review. In the future, more randomized controlled trials (RCTs) including cognitive and motor tasks are needed to provide the evidence on the effect of exercise intervention on brain activity in healthy older adults and people with PD. Furthermore, applying fNIRS to these RCTs is worth investigating, which reduces the effect of noise and allows researchers to explore the motor ability of people with PD and healthy older adults.

## Data Availability

The original contributions presented in the study are included in the article/Supplementary Material; further inquiries can be directed to the corresponding author.
